# Impact of maximum phonation time on postoperative dysphagia and prognosis after cardiac surgery

**DOI:** 10.1016/j.xjon.2024.02.002

**Published:** 2024-02-07

**Authors:** Masato Ogawa, Seimi Satomi-Kobayashi, Mari Hamaguchi, Kodai Komaki, Hifumi Kusu, Kazuhiro P. Izawa, Shunsuke Miyahara, Yoshitada Sakai, Ken-ichi Hirata, Kenji Okada

**Affiliations:** a Department of Rehabilitation Science, Osaka Health Science University, Osaka, Japan; b Division of Rehabilitation Medicine, Kobe University Graduate School of Medicine, Hyogo, Japan; c Department of Public Health, Kobe University Graduate School of Health Sciences, Hyogo, Japan; d Division of Cardiovascular Medicine, Department of Internal Medicine, Kobe University Graduate School of Medicine, Hyogo, Japan; e Division of Cardiovascular Surgery, Department of Surgery, Kobe University Graduate School of Medicine, Hyogo, Japan; f Division of Rehabilitation Medicine, Kobe University Hospital, Hyogo, Japan

**Keywords:** maximum phonation time, deglutition disorders, cardiac surgery, major adverse cardiac and cerebrovascular events, prognosis, frailty

## Abstract

**Objective:**

The incidence of postoperative complications, including dysphagia, increases as the population undergoing cardiovascular surgery ages. This study aimed to explore the potential of maximum phonation time (MPT) as a simple tool for predicting postextubation dysphagia (PED) and major adverse cardiac and cerebrovascular events (MACCEs).

**Methods:**

This retrospective study included 442 patients who underwent elective cardiac surgery at a university hospital. MPT was measured before surgery, and patients were stratified into 2 groups based on normal and abnormal MPTs. Postoperative complications, including PED and MACCEs, were also investigated. Swallowing status was assessed using the Food Intake Level Scale.

**Results:**

MPT predicted PED with prevalence of 11.0% and 18.0% in the normal and abnormal MPT groups, respectively (*P* = .01). During the follow-up period, MACCEs developed in 17.0% of patients. Frailty, European System for Cardiac Operative Risk Evaluation II score, PED, and MPT were markedly associated with MACCEs (adjusted hazard ratios: 2.25, 1.08, 1.96, and 0.96, respectively). Mediation analysis revealed that MPT positively influenced PED and MACCEs, whereas PED positively influenced MACCEs. The trend in restricted cubic spline analysis indicated that the hazard ratio for MACCEs increased sharply when MPT was <10 seconds.

**Conclusions:**

These findings underscore the potential of MPT as a valuable tool in the preoperative assessment and management of patients undergoing cardiac surgery. By incorporating MPT into routine preoperative evaluations, clinicians can identify patients at a higher risk of PED and MACCEs, allowing for targeted interventions and closer postoperative monitoring. This may improve patient outcomes and reduce the health care costs associated with these complications.

## Graphical abstract

**Figure:**
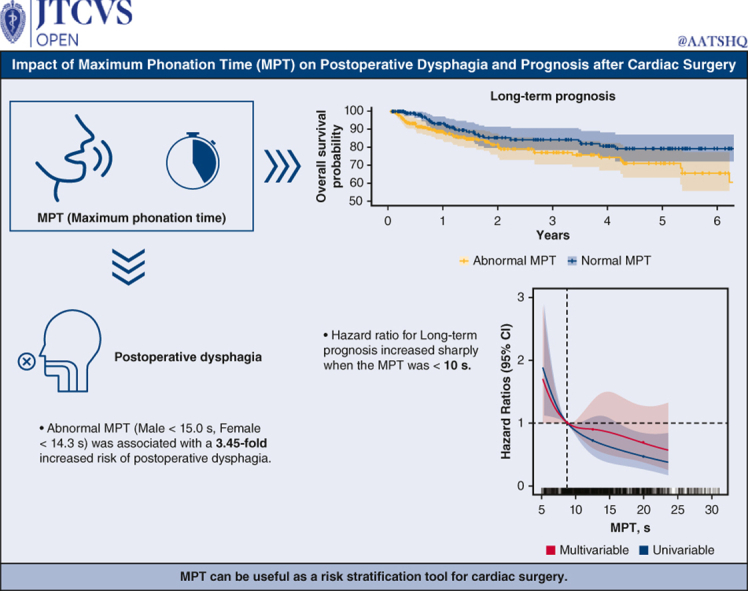

**Figure:**
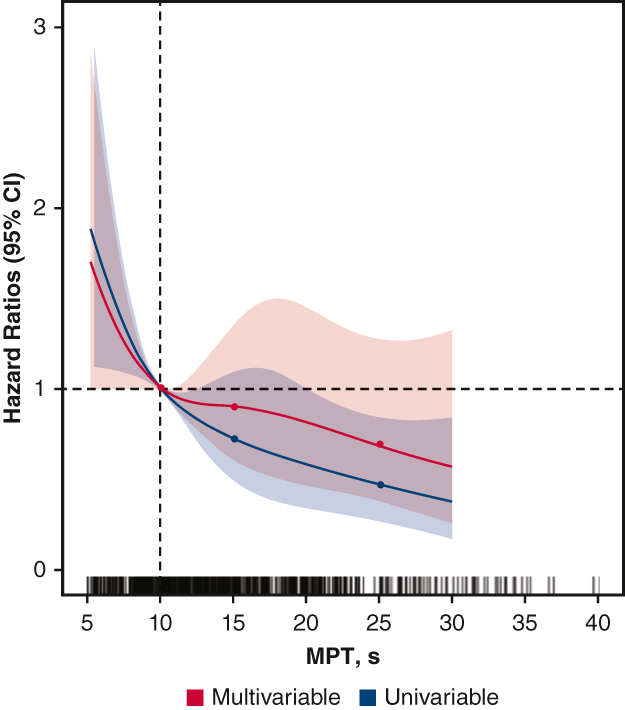
Maximum phonation time predicted long-term prognosis.

**Central Message**

MPT was a potential predictor of postoperative dysphagia. Decreased MPT mediates postoperative dysphagia and is consequently associated with long-term prognosis.

**Perspective**

MPT—which is simple, inexpensive, and easy to measure—can be useful as a risk stratification tool for cardiac surgery.

With the increasing age of the patient population for cardiovascular surgery, the incidence of postoperative complications and less-than-optimal functional recovery has increased, presenting formidable challenges.^1^^,^^2^ Specifically, postextubation dysphagia (PED), characterized as swallowing difficulties following endotracheal extubation, is notably prevalent, with incidence rates reaching up to 15%.^3^^,^^4^ These complications extensively influence both immediate and long-term patient outcomes.^3^^,^^4^ PED is frequently reported and, upon its onset, can lead to complications such as pneumonia.^5^ It also contributes to a prolonged diminution in food intake, physical function, and quality of life.^3^^,^^5^ Such protracted deteriorations in nutritional intake and physical capacity, along with the progression of frailty, influence patient prognosis.^3^ Despite the recognition of frailty as a crucial risk stratification mechanism in older patients undergoing cardiac surgery, there is a conspicuous lack of consensus regarding the most effective approach for precise and efficient perioperative frailty evaluation.^6^ This is further highlighted by the variability in its definition and assessment in the existent literature.^7^^,^^8^ Nevertheless, to minimize postoperative complications, the formulation of pragmatic and efficient assessment methodologies is of paramount importance.

Maximum phonation time (MPT) has been utilized to measure the intensity of dysphonia and verify the effectiveness of speech therapy, owing to its ability to assess bronchial airflow and the operational efficiency of the respiratory mechanism during speech.^9^^,^^10^ Furthermore, MPT has exhibited a positive correlation with exercise tolerance and ventilatory efficiency, along with other pulmonary functions, such as inspiratory and expiratory muscle pressures.^11^, ^12^, ^13^ Despite these promising findings, the potential of MPT as a predictor of postoperative complications and prognostic outcomes in cardiovascular surgery has been largely unexplored in the existing literature. This study aimed to fill this gap by investigating the influence of MPT on PED and prognosis after cardiovascular surgery. MPT may function as an innovative screening test capable of predicting postoperative complications (including dysphagia and aspiration), as well as prognostic outcomes, even in preoperative cardiac patients. Although MPT has been associated with the development of pneumonia during the perioperative phase of esophageal surgery,^14^ its role in the postoperative period of cardiac surgery remains unclear. Therefore, this study presents a novel approach for understanding and predicting the postoperative outcomes of cardiac surgery.

## Materials and Methods

### Study Population

This study was conducted between January 2016 and December 2021 at Kobe University Hospital in Japan. Among the 2278 consecutive patients who underwent cardiac surgery, 442 consecutive inpatients who had undergone elective cardiac surgery and rehabilitation treatment were enrolled. Patients who underwent elective on-pump surgery and subsequently received rehabilitation treatment were considered eligible for inclusion. The exclusion criteria were preoperative dysphagia, severe dementia, missing MPT data, postoperative new-onset stroke, and age younger than 65 years. This study complied with the principles of the Declaration of Helsinki regarding the investigation of humans and was approved by the Kobe University Institutional Review Board (approval No. B200339; approval date: October 7, 2022). Due to the retrospective study design, we used the opt-out method; all participants were notified of their participation in the study and informed that they were free to opt out at any time.

### Clinical Characteristics of the Study Participants

The following baseline characteristics evaluated in this study: age, sex, body mass index, left ventricular ejection fraction, brain natriuretic peptide level, hemoglobin level, comorbidities (such as diabetes, chronic obstructive pulmonary disease, hypertension, dyslipidemia, and chronic kidney disease^15^), medications, New York Heart Association (NYHA) functional class, and nutritional status assessed using the Mini Nutritional Assessment Short-Form.^16^ The European System for Cardiac Operative Risk Evaluation (EuroSCORE) II score was recorded as the operative risk score.^17^ Laboratory data were evaluated within 1 week of cardiac surgery. Perioperative clinical variables recorded included cardiac surgery type, postoperative surgery-related complications, length of intensive care unit stay, length of hospital stay, and discharge location. Frailty was assessed using the Japanese version of the Cardiovascular Health Study frailty index^18^; patients with at least 3 of the following factors were considered physically frail: weakness (handgrip strength), slow gait speed, weight loss, exhaustion, and low physical activity. Physical performance was assessed using the Short Physical Performance Battery (SPPB).^19^

### Assessment of MPT

MPT was measured with a stopwatch while the patient was seated, based on previously reported methods.^9^ Patients were instructed to produce a sustained/a:/vowel sound for as long as possible. MPT was measured within 1 week before surgery and immediately before discharge; three consecutive trials were conducted, and the highest value obtained was used as the MPT index. MPT was measured in increments of 0.1 second, with an upper limit of 60 seconds. A previous study defined an abnormal MPT as <15.0 seconds for men and <14.3 seconds for women.^10^ Patients were stratified into normal and abnormal MPT groups using the same MPT cutoff values.

### Assessments of PED

The swallowing status was assessed using the Food Intake Level Scale (FILS).^20^ Based on the FILS, dysphagia was categorized as no oral intake (score = 1-3), oral intake with alternative nutrition (score = 4-6), and oral intake alone (score = 7-10). The FILS assessment was performed when postoperative oral intake was permitted after the attending cardiologist confirmed that the patient's circulatory status was stable. After postoperative extubation, a well-trained intensive care unit nurse performed bedside swallowing-related evaluation of consciousness level, mouth or tongue function, vocal function, oral hygiene, and cough reflex. Patients with adequate swallowing ability were asked to perform a water swallow test, the results of which were used to determine FILS. We defined dysphagia as a FILS score ≤7, as previously described.^21^

### Follow-up Assessment

The primary end point of this study was to investigate major adverse cardiac and cerebrovascular events (MACCEs) after discharge, which included a composite of death, myocardial infarction, hospital readmission for worsening heart failure or angina, and stroke. All events were based on clinical diagnoses assigned by the treating physician. The patients were followed up as outpatients, and the date and cause of any reported event determined during regularly scheduled outpatient visits were confirmed by reviewing hospital medical records.

### Statistical Analysis

Statistical analyses were performed after confirming normal data distribution using the Shapiro-Wilk test. Patients were stratified into 2 groups based on normal and abnormal MPTs. Differences in baseline clinical characteristics between the groups were determined using independent *t* tests and χ^2^ tests. The results are reported as mean (SD) for parametric data, and median and interquartile range for nonparametric data.

We performed logistic regression analysis to investigate the risk factors for PED; PED incidence was the dependent variable, whereas the clinical characteristics and MPT were independent variables. To analyze the factors influencing MACCEs, multivariate analyses were performed using a Cox proportional hazards regression model. In this analysis, the MACCE incidence was used as the dependent variable, and the independent variables included patient clinical characteristics, MPT, and PED. Kaplan-Meier survival statistics were used to examine the time to the first event, and log-rank analysis was performed to compare MACCEs with and without PED and MPT values.

Restricted cubic spline models were used to assess the relationships between the PED, MACCE, and MPT values. Multiple adjusted splines were adjusted for age, sex, body mass index, type of surgery, Mini Nutritional Assessment Short-Form score, EuroSCORE II score, and NYHA functional class. The splines were restricted to linearity below the first and last knot points. We used the 4 cutoff points for MPT (5.0, 10.0, 15.0, and 25.0 seconds) as knots for the nonlinear effects of continuous MPT assessment. Receiver operating characteristic (ROC) curves were constructed by plotting true-positive rates (sensitivity) against false-positive rates (1 – specificity) to determine the best cutoff value for MPT. The area under the curve (AUC) was calculated from the ROC curve for each variable, and the cutoff value was calculated based on the Youden index.^22^ Mediation analysis was performed to assess PED as a mediator in the relationship between MPT and MACCEs. We hypothesized that PED is a mediator between the MPT value and MACCEs. Thus, 3 pathways were considered: the association between MPT and MACCEs, the association between PED and MACCEs, and the association between MPT and PED.

Mediation (indirect effect) was then established by estimating the direct causal relationship. A bootstrapping method with 5000 resamples was used to calculate the indirect effect and 95% CI. If the 95% CI of the indirect effect did not include 0, the mediation effect was considered significant. The model's goodness of fit was evaluated using the χ^2^ test, comparative fit index, Tucker-Lewis index, and root mean square error of approximation. Sample size was calculated with reference to a previous study,^3^ as well as our unpublished data (power = 0.8, significance level = 0.05, mean difference = 16.0%, n = 600 patients). Statistical analyses were performed using R version 4.3.1 (R Foundation for Statistical Computing) with the *rms*, *lavaan*, and *semTools* packages.

## Results

### Baseline Characteristics

**Figure 1:**
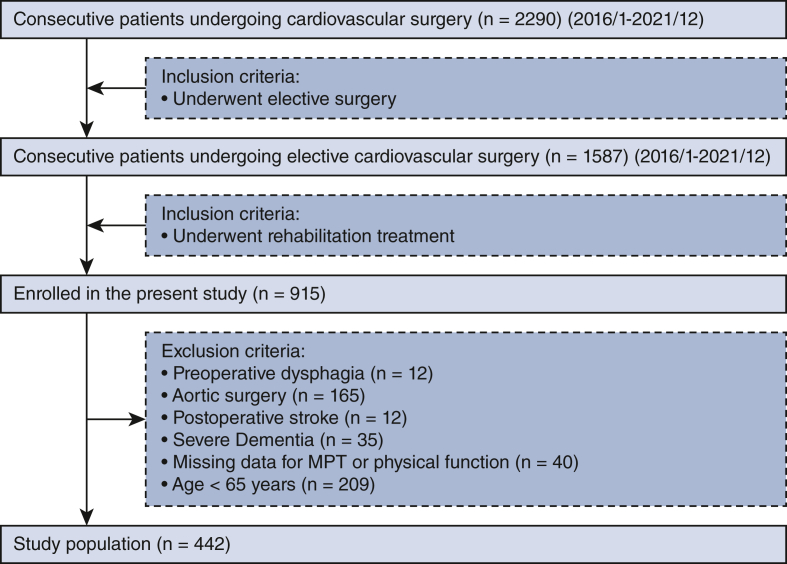
Patient selection flowchart. The flowchart displays the patient flow during the study's enrolment process. *MPT*, Maximum phonation time.

**Figure 2:**
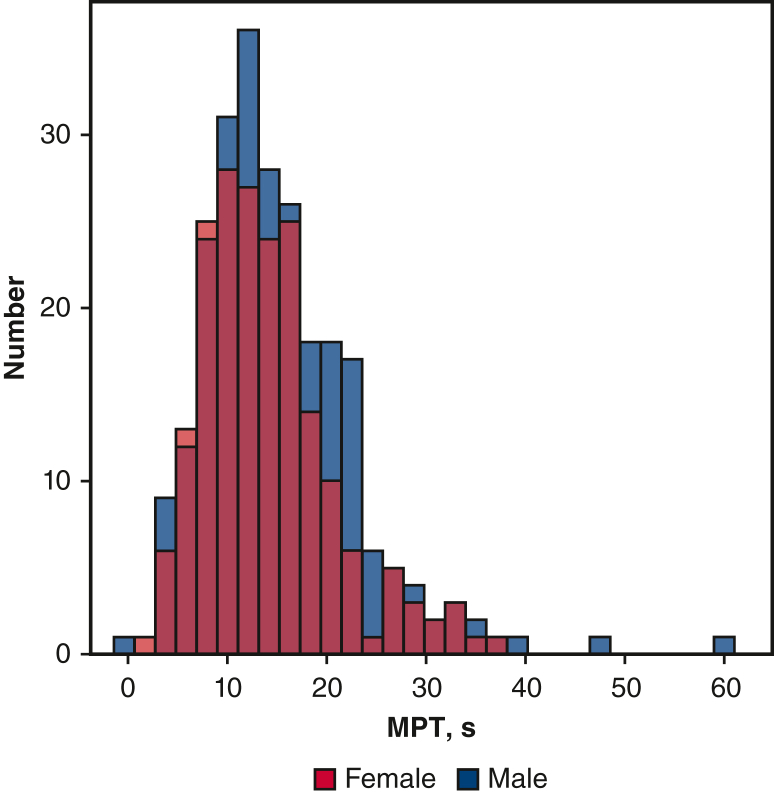
Distribution of maximum phonation time (*MPT*). Preoperative MPT distribution classified by sex.

**Figure E1:**
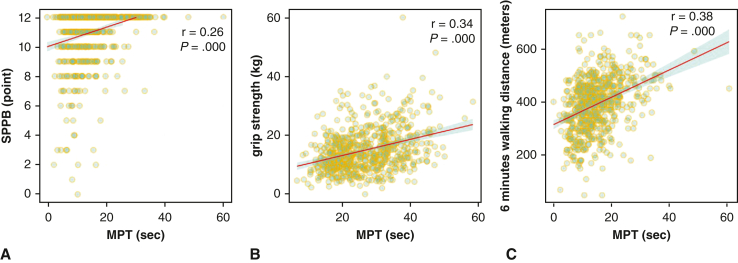
Correlation between maximum phonation time (*MPT*) and physical function. A, Association between MPT and Short Physical Performance Battery (*SPPB*). B, Association between MPT and grip strength. C, Association between MPT and 6-minute walking distance. Physical function positively correlated with MPT.

Of the 2278 consecutive patients, 903 were enrolled in the present study; among them, 442 met the inclusion criteria and 461 were excluded. Of the 461 excluded patients, 209 were younger than age 65 years, 12 had preoperative dysphagia, 35 had severe dementia before surgery, 165 had undergone aortic surgery, and 40 had missing MPT or physical function data (Figure 1). The mean age of the cohort was 71.5 ± 6.4 years, and 44.0% were women. The follow-up period was 2.46 years (interquartile range, 1.62-5.00 years). The mean MPT was 15.0 ± 7.3 seconds, and 45.0% and 55.0% were in the normal and abnormal MPT groups, respectively (Figure 2). The clinical characteristics and between-group differences are presented in Table 1. Compared with patients in the normal MPT group, those in the abnormal MPT group were significantly older and had chronic kidney disease, hypertension, a lower NYHA functional class, and higher malnutrition rates, EuroSCORE II score, pulmonary function, and preoperative frailty (*P* < .05). The rate of postoperative complications was much higher in the abnormal MPT group than in the normal MPT group (31% vs 23%). Mortality was not significantly different between the 2 groups; however, the rates of home discharge were lower in the abnormal MPT group than in the normal MPT group (*P* = .008). Furthermore, physical functions—such as SPPB, grip strength, or 6-minute walking distance—positively correlated with MPT values (Figure E1).

**Table 1:** Clinical and treatment-related characteristics stratified by maximum phonation time (MPT)

Variable	Overall (N = 442)	Abnormal MPT (n = 243)	Normal MPT (n = 199)	*P* value∗
Characteristic				
Age (y)	71.5 ± 6.4	72.8 ± 6.5	69.9 ± 6.0	**<.001**
Female sex	195 (44)	111 (46)	84 (42)	.5
BMI	22.7 ± 3.8	22.7 ± 3.9	22.7 ± 3.8	.9
NYHA functional class				**<.001**
I	82 (19)	25 (10)	57 (29)	
II	208 (47)	107 (44)	101 (51)	
III	152 (34)	111 (46)	41 (21)	
Frail	122 (28)	87 (36)	35 (18)	**<.001**
Laboratory data				
Albumin (g/dL)	3.8 ± 1.0	3.8 ± 1.0	3.8 ± 1.0	.4
BNP (pg/mL)	120.2 (52.0-265.9)	164.0 (72.6-307.9)	83.2 (39.0-194.0)	**<.001**
Hemoglobin (g/dL)	12.9 ± 8.3	13.1 ± 10.8	12.8 ± 2.9	.7
MNA-SF	11.4 ± 2.5	11.2 ± 2.5	11.7 ± 2.3	**.030**
LVEF (%)	57.1 ± 15.3	57.6 ± 14.6	56.5 ± 16.2	.5
MPT (sec)	15.0 ± 7.3	10.1 ± 2.9	21.0 ± 6.4	**<.001**
Comorbidity				
Hypertension	247 (56)	145 (60)	102 (51)	.076
Diabetes mellitus	159 (36)	86 (35)	73 (37)	.8
Chronic kidney disease	124 (28)	83 (34)	41 (21)	**.002**
Dyslipidemia	122 (28)	74 (30)	48 (24)	.14
COPD	128 (29)	80 (33)	48 (24)	**.02**
Physical function				
Grip strength (kg)	27.1 ± 9.2	24.9 ± 8.3	29.7 ± 9.5	**<.001**
SPPB (points)	11.0 ± 1.9	10.6 ± 2.2	11.4 ± 1.4	**<.001**
6-min walking distance (m)	392.6 ± 100.2	367.4 ± 96.4	423.4 ± 96.3	**<.001**
%VC	88.5 ± 16.2	84.7 ± 17.2	92.8 ± 13.7	**<.001**
FEV1 (%)	72.9 ± 10.8	72.4 ± 10.6	74.3 ± 11.0	**.04**
Medications				
B blocker	202 (46)	109 (45)	93 (47)	.7
ACE-I	54 (12)	30 (12)	24 (12)	.9
ARB	131 (30)	72 (30)	59 (30)	.9
Statin	137 (31)	79 (33)	58 (29)	.4
Diuretics	198 (45)	117 (48)	81 (41)	.12
Type of surgery				.8
CABG	58 (9.6)	32 (9.5)	26 (9.7)	
Valve	384 (63)	211 (62)	173 (64)	
Euro SCORE II	6.5 ± 2.4	5.0 ± 4.4	3.6 ± 3.5	.003
Duration of surgery (min)	352.5 ± 101.5	344.4 ± 101.9	362.5 ± 100.2	.062
CPB time (min)	165.5 ± 56.1	161.3 ± 52.0	170.7 ± 60.4	.083
In-hospital complication	119 (27)	77 (31.6)	46 (23.1)	**.027**
PED	43 (9.7)	31 (13)	11 (5.5)	**.010**
Respiratory complications	35 (7.9)	23 (9.5)	12 (6.0)	**.039**
Prolonged ventilation	29 (6.6)	17 (7.0)	12 (6.0)	.7
Pneumonia	22 (5.0)	13 (5.3)	9 (7.6)	.3
Reintubation	7 (1.6)	5 (2.1)	2 (1.0)	.2
Delirium	107 (25)	68 (28)	39 (20)	**.048**
ICU stay (day)	3.7 ± 4.5	3.9 ± 4.4	3.4 ± 4.5	.3
Hospital death	4 (0.9)	3 (1.2)	1 (0.5)	.6
Discharge locations				**.008**
Home	401 (92)	212 (88)	189 (95)	
Transfer	37 (8.4)	28 (12)	9 (4.5)	
Length of hospital stay (day)	23.1 ± 19.3	24.0 ± 15.7	22.0 ± 23.0	.3
MACCE	74 (17.0)	50 (20.6)	24 (12.1)	**.018**

Values are mean ± standard deviation, median (interquartile range), or n (%). Bolded *P*-values indicate *P* < .05. *MPT*, Maximum phonation time; *BMI*, body mass index; *NYHA*, New York Heart Association; *BNP*, brain natriuretic peptide; *MNA*-*SF*, Mini Nutritional Assessment Short-Form; *LVEF*, left ventricular ejection fraction; *COPD*, chronic obstructive pulmonary disease; *SPPB*, Short Physical Performance Battery; *VC*, vital capacity; *FEV1*, forced expiratory volume in 1 second; *B blocker*, beta blocker; *ACE-I*, angiotensin-converting enzyme inhibitor; *ARB*, angiotensin II receptor blocker; *CABG*, coronary aery bypass grafting; *EuroSCORE*, European System for Cardiac Operative Risk Evaluation; *CPB*, cardiopulmonary bypass; *PED*, postextubation dysphagia; *ICU*, intensive care unit; *MACCE*, major adverse cardiac and cerebrovascular event.

∗ Welch's 2-sample *t* test; Pearson χ^2^ test; or Fisher exact test.

### Occurrence of PED and Its Predictors

**Figure 3:**
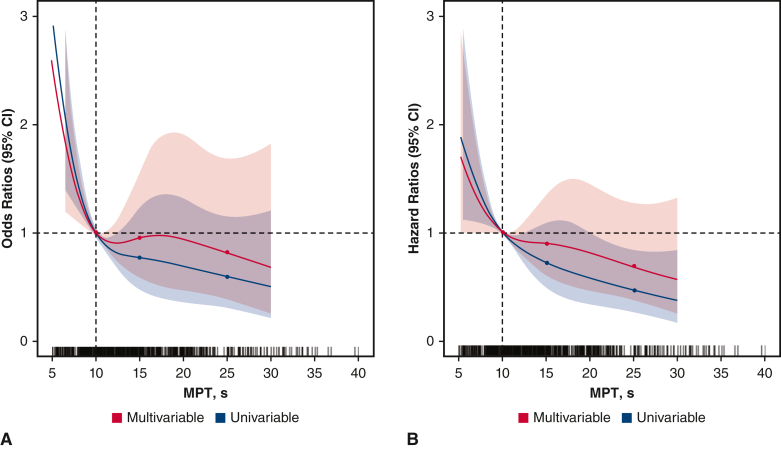
Dose-dependent association between the maximum phonation time (*MPT*), postextubation dysphagia (PED), and major adverse cardiac and cerebrovascular events. The *dotted vertical line* is 10.0, denotes the reference MPT, the *dotted horizontal line* represents an odds or hazard ratio of 1.0, and the *colored areas* represent 95% CIs. *Blue lines* show the univariate model, and *red lines* indicate the multivariable model. Multivariable restricted cubic spline models were adjusted for age, sex, body mass index, New York Heart Association functional class, type of surgery, Japan score, Mini Nutritional Assessment Short-Form, and frailty. A, Association between MPT and the risk of PED. B, Association between body mass index and major adverse cardiac and cerebrovascular events. *CI*, Confidence interval.

**Figure E2:**
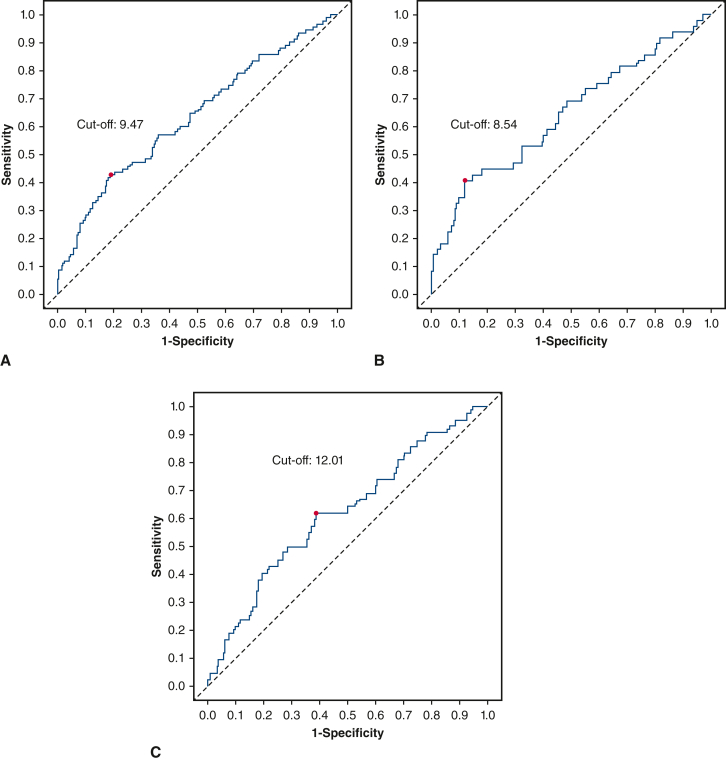
Receiver operator characteristic curve for maximum phonation time (MPT) receiver operating characteristic curve for MPT predicting the incidence of postoperative dysphagia. A, Total cohort. The MPT cutoff was set at 9.5 seconds for an increased risk of developing postextubation dysphagia (PED) (area under the curve [AUC], 0.63; *P* < .001). B, Results in male patients. The MPT cutoff was set at 8.4 seconds for an increased risk of developing PED (AUC, 0.62; *P* = .001). C, Results in female patients. The MPT cutoff was set at 12.0 seconds for an increased risk of PED development (AUC, 0.64; *P* = .0016).

**Figure E3:**
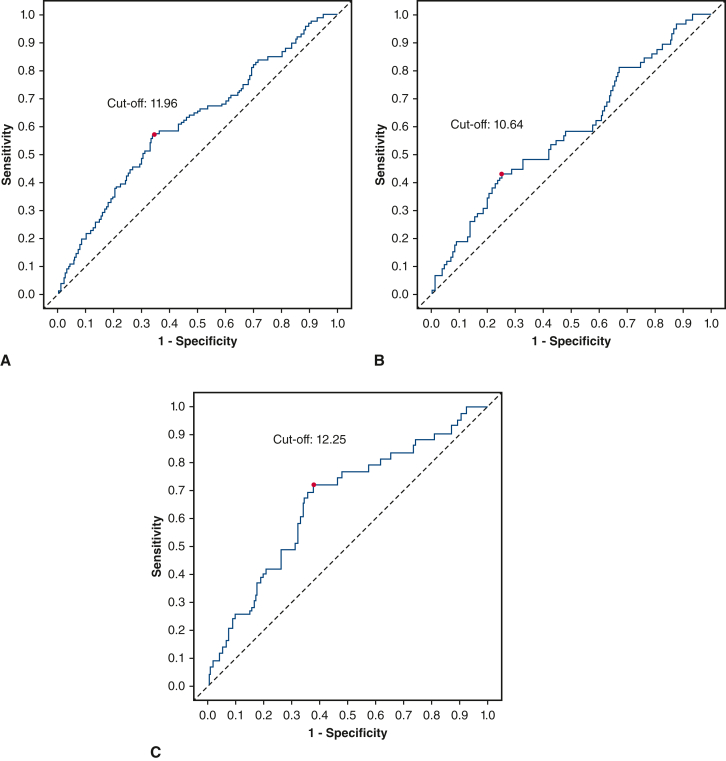
Receiver operator characteristic curve for maximum phonation time (MPT). Receiver operating characteristic curve for MPT predicting the incidence of major adverse cardiac and cerebrovascular events (MACCEs). A, Total cohort. The MPT cutoff was set at 12.0 seconds for an increased risk of MACCE development (area under the curve [AUC], 0.61; *P* = .0003). B, Results in male patients. The MPT cutoff was set at 10.6 seconds for an increased risk of MACCE development (AUC, 0.58; *P* = .028). C, Results in female patients. The MPT cutoff was set at 12.2 seconds for an increased risk of MACCE development (AUC, 0.66; *P* = .0010).

The prevalence of PED was 5.5% and 13.0% in the normal and abnormal MPT groups, respectively (*P* = .010). The results of the multivariate logistic analyses for PED are shown in Table 2. In the multivariate analysis, frailty (adjusted odds ratio, 5.53; 95% CI, 2.53-12.1) and MPT (adjusted odds ratio, 0.96; 95% CI, 0.92-0.99) were independently associated with PED development after adjusting for potential confounders. The restricted cubic spline showed a trend of increasing PED risk as MPT decreased (Figure 3, *A*). The risk of PED increased markedly, particularly when MPT was <10 seconds. The cutoff value for MPT determined by the ROC curve analysis was 9.5 seconds (AUC = 0.63; *P* < .0001) for the entire cohort, 12.0 seconds for men, and 8.5 seconds for women (Figures E2 and E3). The abnormal MPT group showed a higher risk of in-hospital complications, which are predominantly respiratory complications, with marginal statistical significance in multivariate analysis (*P* = .051) (Table E1). On the other hand, there was no sex difference in the predictive ability of MPT for PED.

**Table 2:** Multivariate analysis of predictive factors for the development of postextubation dysphagia

Characteristic	Odds ratio	95% CI	*P* value
Age	1.00	0.97-1.065	.632
Male sex	1.23	0.62-2.45	.556
BMI	0.93	0.83-1.04	.210
Type of surgery: Valve	0.74	0.29-1.93	.544
NYHA functional class	0.62	0.35-1.09	.090
MPT	0.95	0.89-0.98	**.034**
Frail	5.44	2.47-11.9	**<.001**
MNA-SF	0.98	0.84-1.14	.790
EuroSCORE II	1.08	1.01-1.18	**.012**

Bolded *P*-values indicate *P* < .05. *CI*, Confidence interval; *BMI*, body mass index; *NYHA*, New York Heart Association; *MPT*, maximum phonation time; *MNA-SF*, Mini Nutritional Assessment Short-Form; *EuroSCORE*, European System for Cardiac Operative Risk Evaluation.

### Results for MACCEs

**Figure 4:**
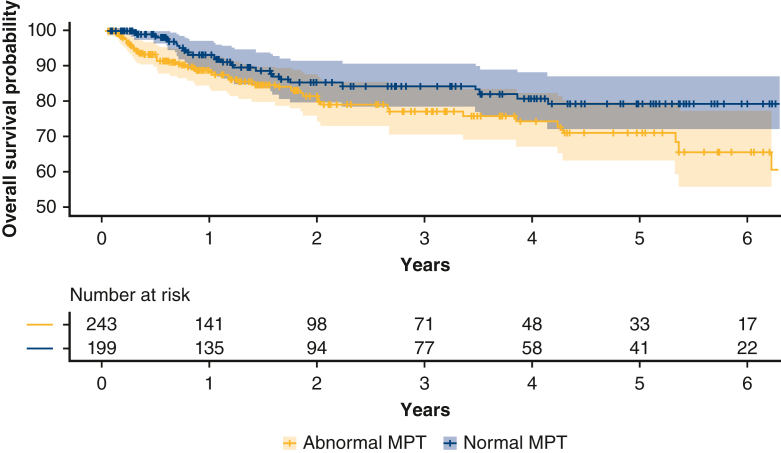
Time to the major adverse cardiac and cerebrovascular events Time to the major adverse cardiac and cerebrovascular events Kaplan-Meier curves showing major adverse cardiac and cerebrovascular event rates between the normal (*blue line*) and abnormal (*yellow line*) maximum phonation time (*MPT*) groups (log-rank test *P* = .009). The *solid lines* represent the estimates, and the surrounding bands are 95% CI.

**Figure E4:**
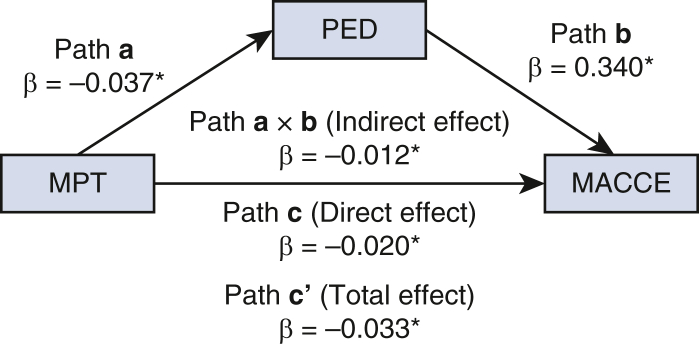
Results of mediation analysis between maximum phonation time (*MPT*) and major adverse cardiac and cerebrovascular events (*MACCEs*). Postextubation dysphagia (*PED*) is a mediation variable. ∗*P* < .05. ∗∗*P* < .01.

During the follow-up period, 17.0% of the patients developed MACCEs. Overall, 74 patients (17.0%) had MACCEs (heart failure, n = 51; stroke, n = 12; nonfatal myocardial infarction, n = 3; and death, n = 8). Additionally, heart failure was commonly accompanied by complications of pneumonia. Table 3 shows the results of multivariate analyses using the Cox proportional hazards regression model to predict MACCEs. The multivariate Cox proportional hazard regression analysis showed that frailty (hazard ratio [HR], 2.42; 95% CI, 1.35-4.33; *P* = .003), EuroSCORE II (HR, 1.08; 95% CI, 1.01-1.10; *P* = .007), PED (HR, 1.88; 95% CI, 1.04-3.37; *P* = .035) and MPT (HR, 0.96; 95% CI, 0.93-0.99; *P* = .04) were significantly associated with MACCEs after covariate adjustment. The Kaplan-Meier analysis revealed that the abnormal MPT group had a worse prognosis than the normal MPT group (*P* = .00 l) (Figure 4). The restricted cubic spline showed an increasing trend of MACCE risk as MPT decreased (Figure 3, *B*). The results of the mediation analysis are shown in Figure E4 and Table E2. The model fit was acceptable (comparative fit index = 0.974, Tucker-Lewis index = 0.952, root mean square error of approximation = 0.048),^23^ and the mediation analysis revealed that MPT positively influenced PED (β = 0.150; *P* = .030) and MACCEs (β = 0.249; *P* = .002). PED also positively influenced MACCEs (β = 0.189; *P* = .026), and MPT indirectly influenced MACCEs by influencing PED (indirect effect, 0.028; 95% CI, 0.001-0.078). The total effect of the association between MPT and MACCEs was statistically significant (indirect effect, 0.028; 95% CI, 0.001-0.078). The cutoff value for the MPT determined by the ROC curve analysis was 11.9 seconds (AUC = 0.61; *P* = .003) for the entire cohort, 10.6 seconds for men, and 12.1 seconds for women.

**Table 3:** Cox proportional hazard regression analysis of predictive factors for major adverse cardiac and cerebrovascular event development

Characteristic	Hazard ratio	95% CI	*P* value
Age	1.01	0.98-1.03	.613
Male sex	1.16	0.70-1.87	.528
BMI	0.99	0.93-1.07	.717
Type of surgery: Valve	2.82	0.88-9.19	.080
NYHA functional class	1.02	0.69-1.50	.912
MPT	0.96	0.92-0.99	**.041**
Frail	2.25	1.25-4.05	**.007**
EuroSCORE II	1.08	1.01-1.10	**.007**
PED	1.96	1.10-3.51	**.023**

Bolded *P*-values indicate *P* < .05. *CI*, Confidence interval; *BMI*, body mass index; *NYHA*, New York Heart Association; *MPT*, maximum phonation time; *EuroSCORE*, European System for Cardiac Operative Risk Evaluation; *PED*, postextubation dysphagia.

## Discussion

**Figure 5:**
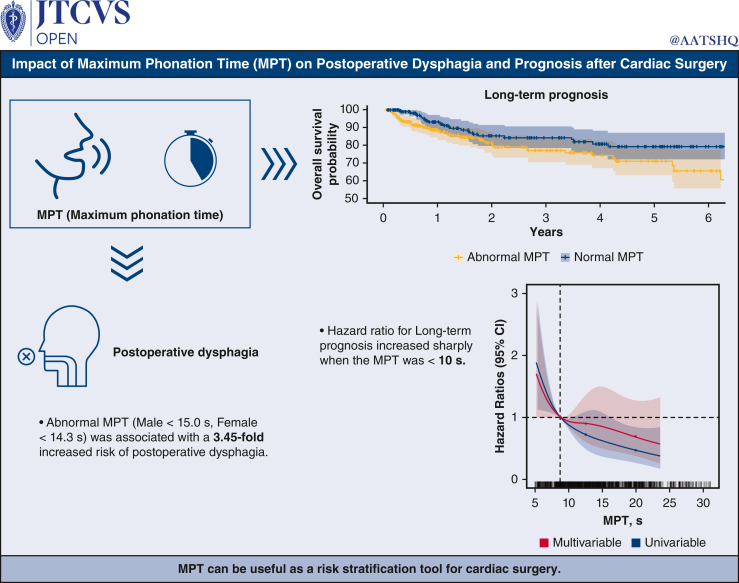
Graphical abstract. Influence of maximum phonation time (*MPT*) on postoperative dysphagia and prognosis after cardiac surgery.

The present study revealed 2 remarkable findings that contribute to our understanding of postoperative outcomes of cardiac surgery. First, MPT emerged as a potential predictor of PED. Second, our study demonstrated the predictive value of MPT for MACCEs. Furthermore, decreased MPT was found to mediate PED and was consequently associated with MACCEs. These findings underscore the potential of MPT as a valuable tool in the preoperative assessment and management of patients undergoing cardiac surgery (Figure 5).

The mean MPT in our study was 15.0 ± 7.3 seconds. Studies investigating MPT are sparse. MPT can vary widely among individuals, with adult men and women typically being able to sustain vowel sounds for 25 to 35 seconds and 15 to 25 seconds, respectively.^24^ Another study demonstrated that age is a significant factor influencing maximum speech performance.^25^ To the best of our knowledge, this study is the first to report MPT results in an older surgical patient population. Although our findings slightly differ from those of previous research, our study provides valuable insights into the potential of MPT as a predictive tool that considers the characteristics of cardiac surgical patients, including age and comorbidities.

We found that MPT could serve as a predictor of PED, with a prevalence of 5.5% and 13.0% in the normal and abnormal MPT groups, respectively. This finding aligns with previous research identifying MPT as a predictive factor for postoperative complications—such as aspiration pneumonia^26^ and dysphagia—in patients undergoing occipital spinal fusion.^27^ We previously reported that even transient PED lowers functional status and worsens the long-term prognosis, strongly indicating the usefulness of the MPT.^3^ Given the substantial negative influence of PEDs, even when transient, MPT has been demonstrated to be very effective in risk stratification. MPT is a comprehensive assessment of respiratory, swallowing, and speech functions, as well as motor tolerance. It may also identify subtle declines in these abilities that were not recognized preoperatively. It is possible that this subtle decline in abilities may have manifested itself in the form of dysphagia due to postoperative invasion. Incorporating MPT into the evaluation of frailty regardless of sex could prognosticate postoperative complications, particularly dysphagia, and potentially enhance the precision of predicting outcomes. Delirium notably emerged as a more prevalent complication in the abnormal MPT groups. Considering the established increased vulnerability of frail individuals to delirium,^28^ the incorporation of MPT in evaluating frailty is both justified and advantageous.

MPT was identified as a predictor of MACCEs, with 17.0% of the patients developing MACCEs during the follow-up period. This finding is novel, as there is a lack of studies that directly support MPT as a predictor of MACCEs; however, MPT has been recognized as a useful assessment tool for older adults requiring long-term care/support,^29^ which may indirectly suggest its relevance in predicting adverse health outcomes in this population. The trend in the restricted cubic spline analysis indicated that the HR for MACCEs increased sharply when MPT was <10 seconds. Furthermore, mediation analysis revealed that MPT indirectly influenced MACCEs by influencing PED. MPT exhibits a robust correlation with physical functionality and effectively delineates a specific facet of frailty in detail. It is well known that frailty serves as a predictor for heart failure,^30^ a finding that aligns with the results observed in this study. It seems likely that the decrease in preoperative reserve may worsen the long-term prognoses of patients via respiratory complications and PED. PED is often transient, with a 73% improvement occurring during hospitalization. However, there remains a risk of further functional decline and impaired physical function recovery.

Our findings have profound and widespread clinical implications. The identification of MPT as a predictor of PED and MACCEs may revolutionize preoperative assessments and postoperative care in cardiovascular surgery. By incorporating MPT into routine preoperative evaluations, clinicians can identify patients at a higher risk of PED and MACCEs, allowing for targeted interventions and closer postoperative monitoring. Furthermore, enhancing MPT, which is linked to physical function, might be possible through prehabilitation.^31^ Recently, a multidimensional prehabilitation approach, which includes nutritional intervention, physical rehabilitation, and psychological care, has been recognized as an effective strategy to improve postoperative outcomes for cardiac surgery.^32^ Postoperative management, including assessing swallowing function, promptly determining the right nutrition, dose, and administration methods, and starting early physical and respiratory rehabilitation, could also offer insights into better outcomes. This could potentially improve patient outcomes and reduce the health care costs associated with these complications. Our findings may stimulate further research on the underlying mechanisms linking MPT, PED, and MACCEs, potentially uncovering new therapeutic targets.

### Study Limitations

Despite the remarkable contributions of our study, it has some limitations. First, it was conducted at a single center, which may have limited the generalizability of our findings. Second, our study was observational in nature, which precluded us from establishing causal relationships between MPT, PED, and MACCEs. The applicability of the findings requires caution, particularly due to the study's observational nature and its exclusive inclusion of Asian participants. Further multicenter studies are required to validate our findings in different populations and settings. Third, the FILS score was used to assess PED as an indirect assessment of the swallowing function of food substances. Subsequently, there remain unanswered questions concerning mechanical injuries, such as vocal cord immobility or arytenoid cartilage dislocation. Fourth, videofluoroscopy or videoendoscopy is the gold standard for diagnosing dysphagia. However, these evaluations were not possible in the present study due to their invasive nature, patient discomfort, and radiation risks. In addition, the effectiveness of management based on videoendoscopy or videofluoroscopy in intensive care units remains unclear.^33^ Further investigation is required to develop a precise and clinically viable method for swallowing assessment that is also sustainable. Lastly, our study did not include an investigation of the temporal extent of PED, which prevented us from gaining understanding into the protracted trajectory of PED recovery. Similarly, the longitudinal assessment of MPT remains unexplored, inhibiting discourse on its long-term progression.

Despite these limitations, our study has several strengths. This is the first study to identify MPT as a predictor of MACCEs, filling a significant gap in the literature. Our study also highlights the potential role of PED as a mediator in the relationship between MPT and MACCE, providing a new perspective of these complex relationships. Our findings thus have important clinical implications for improving patient outcomes and guiding future research in this field.

## Conclusions

This study demonstrated that preoperative MPT was strongly associated with swallowing dysfunction in patients who underwent cardiac surgery. MPT predicts long-term prognosis and the occurrence of PED worsens patient prognosis. Notably, functional decline accompanied by PED was a powerful predictor of poor prognosis 3 years postoperatively. MPT—which is simple, inexpensive, and easy to measure—can be useful as a risk stratification tool for cardiac surgery.

## Conflict of Interest Statement

The authors reported no conflicts of interest.

The *Journal* policy requires editors and reviewers to disclose conflicts of interest and to decline handling manuscripts for which they may have a conflict of interest. The editors and reviewers of this article have no conflicts of interest.

## Figures and Tables

**Table E1:** Multivariable analysis of predicting factors for the development of in-hospital complications

Characteristic	Odds ratio	95% CI	*P* value
Age	1.01	0.95-1.03	.568
Male sex	0.86	0.55-1.33	.488
BMI	0.98	0.92-1.04	.513
Type of surgery: Valve	1.76	0.83-3.71	.140
NYHA functional class	1.40	1.02-2.12	**.041**
MPT	0.97	0.94-1.01	.051
Frail	2.48	1.23-5.12	**.028**
MNA-SF	0.97	0.88-1.04	.333
EuroSCORE II	1.06	1.01-1.12	**.039**

Bolded *P*-values indicate *P* < .05. *CI*, Confidence interval; *BMI*, body mass index; *NYHA*, New York Heart Association; *MPT*, maximum phonation time; *MNA-SF*, Mini Nutritional Assessment Short-Form; *EuroSCORE*, European System for Cardiac Operative Risk Evaluation.

**Table E2:** Testing the mediation effect of postextubation dysphagia (PED) between maximum phonation time (MPT) and major adverse cardiac and cerebrovascular event (MACCE)

Path	Effect	β	SE	*P* value
a	MPT→PED	−0.037	0.009	.0002
b	PED→MACCE	0.340	0.081	.001
c	(Direct effect) MPT→MACCE	−0.020	0.010	.040
c’	(Total effect) MPT→MACCE	−0.033	0.010	.001
a × b	(Indirect effect) MPT→MACCE	−0.012	0.004	.003

*SE*, Standard error; *MPT*, maximum phonation time; *PED*, postextubation dysphagia; *MACCE*, major adverse cardiac and cerebrovascular event.
